# The Role of RASs /RVs in the Current Management of HCV

**DOI:** 10.3390/v13102096

**Published:** 2021-10-18

**Authors:** Konstantinos Malandris, Georgios Kalopitas, Eleni Theocharidou, Georgios Germanidis

**Affiliations:** 1 Second Department of Internal Medicine, Hippokration General Hospital, Aristotle University of Thessaloniki, 54642 Thessaloniki, Greece; kostas_malandris@yahoo.gr (K.M.); elenit@auth.gr (E.T.); 2 First Department of Internal Medicine, AHEPA University Hospital, Aristotle University of Thessaloniki, 54636 Thessaloniki, Greece; gekalopi@auth.gr; 3 Basic and Translational Research Unit, Special Unit for Biomedical Research and Education, School of Medicine, Faculty of Health Sciences, Aristotle University of Thessaloniki, 54636 Thessaloniki, Greece

**Keywords:** HCV, viral resistance, DAA, RAS

## Abstract

The approval of combination therapies with direct-acting antiviral (DAA) regimens has led to significant progress in the field of hepatitis C virus (HCV) treatment. Although most patients treated with these agents achieve a virological cure, resistance to DAAs is a major issue. The rapid emergence of resistance-associated substitutions (RASs), in particular in the context of incomplete drug pressure, has an impact on sustained virological response (SVR) rates. Several RASs in NS3, NS5A and NS5B have been linked with reduced susceptibility to DAAs. RAS vary based on HCV characteristics and the different drug classes. DAA-resistant HCV variant haplotypes (RVs) are dominant in cases of virological failure. Viruses with resistance to NS3-4A protease inhibitors are only detected in the peripheral blood in a time frame ranging from weeks to months following completion of treatment, whereas NS5A inhibitor-resistant viruses may persist for years. Novel agents have been developed that demonstrate promising results in DAA-experienced patients. The recent approval of broad-spectrum drug combinations with a high genetic barrier to resistance and antiviral potency may overcome the problem of resistance.

## 1. Introduction—Basic Concepts

HCV has an estimated half-life that ranges from 2 to 5 h with a high turnover rate. More specifically, 10^10^–10^12^ virions are produced and excreted daily in an infected patient.

The increased error rate of the HCV-RNA-dependent RNA polymerase combined with the increased virion production (100-fold higher than that of human immunodeficiency virus) [1] leads to a complex composition of viral genetic populations named “quasi-species”. Quasispecies represent an evolving mixture of mutant haplotypes that share an increased sequence similarity and preexist inside an infected individual before the initiation of treatment [2].

Viral quasispecies are presently defined as assortments of firmly related viral genomes exposed to a non-stop process of genetic variation, competition among the created variants and selection of the most suitable forms for a given environment [2]. Quasispecies diversity has potential implications for liver disease progression, responses to antiviral therapy and vaccine development. A major aspect of the quasispecies concept is the continuous interaction between individual viruses that leads to an equilibrium of the different variants through both positive and negative interplay. Virus adaptability is inextricably linked with a mutant viral spectrum of varying composition. Mutant spectrum-mediated adaptability covers three parameters: (I) the amount of mutants present for a given time point in the quasispecies, (II) the various haplotypes and (III) the amount of viral particles in the population of interest [3].

Fitness is defined as the ability of a virus to produce infectious progeny. Subsequently, it reflects the intrinsic ability of a virus to adapt in a given environment. The continuous replication of large viral particles in a given environment leads to fitness acquisition and adaptation, whereas repeated bottlenecks that result in the accumulation of mutations lead to the opposite direction. The direct association between fitness and viral load has been linked to disease progression and responses to antiviral regimens [3].

The existence of various diversity indices is of great importance in order to define viral quasispecies at a molecular level [4]. A parameter that is able to predict the degree of adaptability of the quasispecies in a given environment is complexity. The complexity of the mutant spectrum is a predicting factor for disease progression, as well as response to treatments. A reduction in the complexity of the mutant spectrum indicates major events, more specifically the occurrence of a pervasive selection episode or a decrease in the viral population. An antiviral mutagen that is considered effective in a lethal mutagenesis design should lead to an increase in the complexity of the mutant spectrum, at least in a transient way. Higher quasispecies complexity has been demonstrated in patients with more advanced liver disease and those with hepatocellular carcinoma [5]. The information needed to obtain viral quasispecies complexity estimates is located in the multiple alignment of all unique sequences (haplotypes) that fully cover the region of interest (amplicon) and their observed frequencies [4]. Multiple haplotypes aligned together indicate the entities present in viral quasispecies. The frequencies provide information on the plethora of those entities and are tightly related to the fitness of each haplotype. Each diversity index provides a fragmental view of the information for the viral quasispecies [4].

Quasispecies’ genetic diversity is estimated to be in the range of 1–3% and affects mainly structural rather than non-structural proteins [6]. Genomic sequencing has demonstrated the presence of eight HCV genotypes (1–8) and 105 subtypes that differ in nucleotide sequence by 30% and 15%, respectively [7]. A major concern in viral quasispecies is how to correlate genetic with phenotypic and functional diversity.

All functional indices that are based on genetic distances assume that genomes that are far apart in sequence space are more likely to have functional differences compared to sequences that are close together. This assumption is not fully accepted for viral quasispecies since single mutations in genomes can influence pivotal functional aspects such as resistance against the immune system or antiviral agents [4]. A major drawback in the assessment of the virus population complexity for biological inferences is that viral quasispecies share a variable profile. The relative frequency of each new haplotype is related to the previous and current fitness levels of the associated genomes, in interaction with other members of the mutant ensemble. Subsequently, contractions and expansions of diversity can occur. Diversity contraction may result from the emergence of a new haplotype that shares increased fitness compared to the existing mutants, leading eventually to an increase in the viral load accompanied by a temporary reduction in complexity. Fluctuations in fitness among haplotypes may arise from environmental changes, the production of new mutants, or both. The type of mutation is another major concern. The increased number of point mutations plays a significant role in the study of lethal mutagenesis induced from treatment with virus-specific nucleotide analogues, as each mutagenic nucleotide analogue has a predilection for certain mutation types and an accurate estimation of the types of mutation is suggestive for the presence or absence of mutagenic activity. The degree of mutagenic activity is of paramount importance in the everyday clinical setting where the use of antiviral agents with different mechanisms of action is extensive. The selection of appropriate diversity indices leads to an adequate description of viral quasispecies [4].

**Figure 1:**
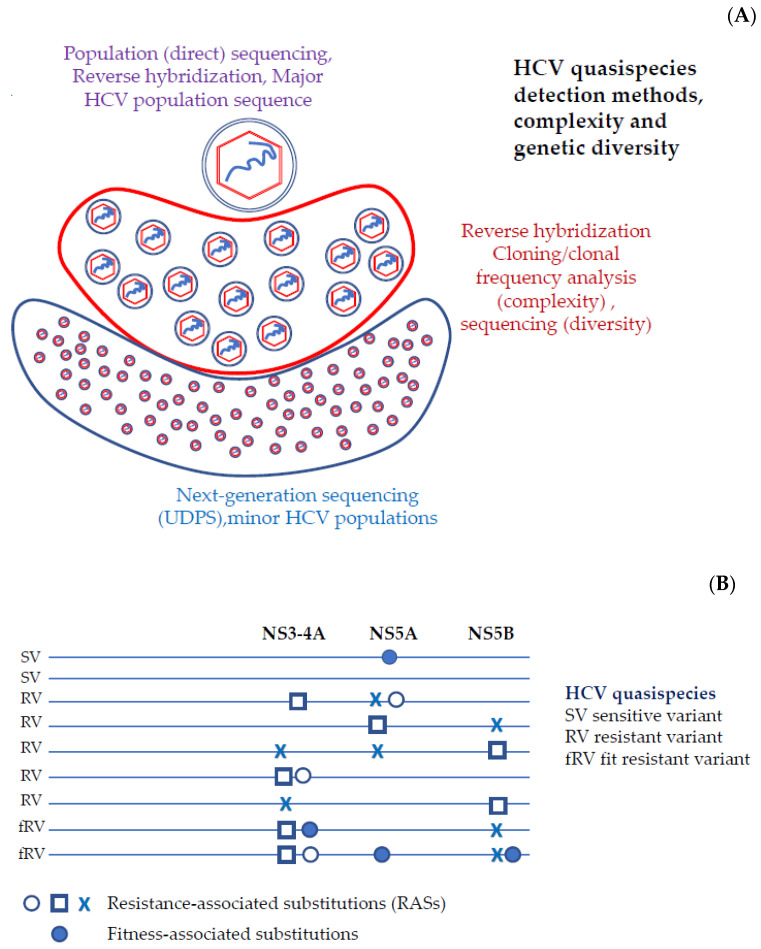
(**A**) The “cloud” of HCV quasispecies in the space of sequences; Mixture of virions that are genetically distinct but closely related. The assay methods for the detection of major populations should report the presence of RASs with a validated and repeatable *sensitivity of 15%,* equivalent to *population sequencing*. The number of different variants and their relative frequency give estimated measures of complexity and the genetic distances measures of diversity. (**B**) Viral variants are individual *full-length viruses* that constitute the HCV quasispecies in a patient. Resistant variants contain one or several *RASs*, which are *single amino acid changes* that reduce susceptibility to a *DAA* or a class of *DAAs*. *Fitness associated substitution(s)* are single amino acid changes that do not alter DAA susceptibility but increase the fitness of the resistant variants.

With the term classical indices, we refer to the indices used to describe viral quasispecies through the nucleotide sequencing process. Prior to the New Generation Sequencing, the description of the viral quasispecies was performed with the cloning and amplification of DNA or RNA, followed by Sanger sequencing. The information for deriving an estimate for the complexity of the viral quasispecies is located in the haplotypes that fully cover the amplicon and their observed frequencies (Figure 1A).

In summary, indices from ecology can be used in order to characterize the complexity of viral quasispecies. However, the concept of complexity is multifaceted and subsequently no single index can describe in depth the actual meaning [4].

The availability of a cell culture system for HCV has enabled the generation of HCV that exhibits different levels of fitness as a result of several passages in human hepatoma cells [8,9]. One of the studies’ conclusions is that replicative fitness can be an independent prediction factor of the response of HCV to inhibitors employed in therapy. Interferon and pegylated interferon exert their antiviral action mainly by enhancing the host-specific antiviral immune response and help clear the infected hepatocytes during the slow second phase of viral HCV kinetics [10]. There is the theoretical concern that HCV variants may emerge under the immunological pressure (bottleneck) exerted by interferon. However, research in pre- and post-interferon samples did not show the emergence of any clinically relevant RAS that may confer resistance to DAAs [11]. It is well known that viruses that have never been exposed to inhibitors can in fact have mutations that produce inhibitor resistance [8,9]. Subsequently, in the HCV studies, it was crucial to exclude the inhibitory mutations that were not acquired through fitness gain. This was made possible by NGS, the multiplicity of infection (MOI)-independent kinetics of virus production in the presence of inhibitors and the maintenance of the resistance phenotype in biological clones of the passaged populations [9]. In other words, the response of a HCV infected patient to a treatment can be influenced by the evolutionary history of the virus. More specifically, the long-term replication of the virus in a patient with HCV infection can promote viral fitness to the detriment of treatment efficacy, as observed in clinical practice.

## 2. HCV Resistance to DAAs

DAA therapy confers overall SVR rates above 90% [12]. Although only a small proportion of optimally treated patients (2–5%) fail DAA therapy, this translates into a significant absolute number of patients that require retreatment given the prevalence of HCV worldwide. Factors that have been associated with DAA failure include adequacy of treatment (optimal combination and duration), resistance profile (mainly RAS to NS5A) and baseline parameters such as genotype/subtype (higher risk of failure in genotypes 1a and 3), disease severity (cirrhosis) and previous treatment exposure [13].

Wild-type amino acids differ across the various HCV types (geno/subtype and geographic origin) [1]. Geno2Pheno was used to define the reference amino acid sequence for each HCV genotype [14]. Any alteration in the reference amino acid sequence that leads to increased resistance of a virus to one or more antiviral drugs is defined as resistance-associated substitution (RAS) [15,16,17]. These are amino acid substitutions that are able to impair and reduce the potency of DAAs either in vitro or in vivo. A resistant variant (RV) haplotype is characterized by the presence of RASs. In the context of HCV quasispecies, it is likely that different variants with multiple amino acid substitutions that confer drug resistance may coexist within the same host (Figure 1B). A certain substitution can lead to a phenotypic reduction in susceptibility to one or more antiviral agents, although there are substitutions that are not associated with drug resistance. A drug-specific RAS is defined as the substitution that leads to the reduction in the susceptibility of a virus for a certain drug, whereas drug-class RASs are substitutions that reduce the susceptibility of a virus to at least one member of a drug-class [15,16,17]. According to the latest international guidelines, a RAS is defined by the following: the HCV type (geno/subtype); the amino acid position; and the HCV protein [15,16,17]. Each RAS is described by a capital letter suggestive of the reference amino acid, a number indicating the position of the amino acid in the wild-type protein and a second letter suggesting the amino acid that is actually found in the sequence of interest.

The emergence and type of RAS depends upon the genotype/subtype and the drugs to which the virus has been exposed [18]. RAS is more common in genotypes 1a and 3 compared to other genotypes [19]. It can emerge as a result of exposure to DAA therapy but can also exist in DAA-naïve patients. RAS in the NS3 and NS5A genes is present in up to 50% and 15%, respectively, in DAA-naïve patients [20]. Non nucleotide NS5B RAS is present in up to 30%, whereas nucleotide NS5B RAS is rare (1–3%), likely due to its adverse impact on viral fitness [21].

The risk of RAS is higher in the context of virologic breakthrough during DAA therapy as opposed to relapse following DAA treatment [19]. The duration of treatment is also relevant, as patients who experience relapse with long regimens are more likely to harbor RAS-associated variants, whereas those who relapse after short regimens are more likely to relapse with wild-type strains [19]. RAS that is present prior to treatment might become more potent and, therefore, clinically relevant after exposure to DAA [22]. It is also possible that RAS present <15% at baseline can be selected as a majority variant following treatment [23]. Previous studies have suggested that an increase in viral fitness rather than isolated RAS may account for resistance to treatment and treatment failure. Viral fitness may increase as a result of prolonged HCV replication and RAS accumulation [9]. Patients with prolonged infection and more advanced liver disease may, therefore, exhibit higher resistance to DAA therapy as a result of increased viral fitness.

Genotypic analyses include next-generation sequencing (NGS) and population sequencing. Their main difference arises from their ability to detect single specific substitutions within the viral quasispecies of interest. Population sequencing is able to detect variants that represent over 15–20% of the quasispecies, whereas NGS can reach detection rates of 0.1–1%. Nevertheless, existing data suggest that only RASs present in >15% are clinically meaningful. Therefore, in clinical practice, HCV genotypic resistance testing can be carried out with population sequencing. If NGS is applied, a cut-off value of 15% for reporting RASs is recommended [15]. Drug-specific RASs that are detected with NGS might not promote significant resistance and decrease SVR rates with existing DAA regimens [15,16,17].

A recent systematic review of RAS sequencing protocols, alongside a public library of sequencing primers, provided guidance in the field of RASs screening and identification. Experts have identified several limitations of the existing methods and have highlighted areas requiring further development [24].

## 3. NS3-4A Protease Inhibitors

NS3 protease inhibitors (PIs) are peptide-like inhibitors that compete with the intrinsic NS3 serine-protease substrates, thus preventing the viral polyprotein cleavage [25]. With the exception of the catalytic triad, only 47% of NS3-residues are fully conserved across HCV genotypes (GTs) [26], making it difficult to design pangenotypic inhibitors. NS3 protease RASs are commonly found at the baseline. The most frequently detected RAS is Q80K, found approximately in 13.6%, mainly in patients with GT1a infection. The substitution A156T is associated with increased resistance, but is not detected at baseline [15]. In patients with HCV genotype 1b, common high-level RAS include R155K and D168E [18]. The most prevalent RAS in genotype 3 is Y93H, which is commonly detected in patients who fail to achieve SVR [27].

**Figure 2:**
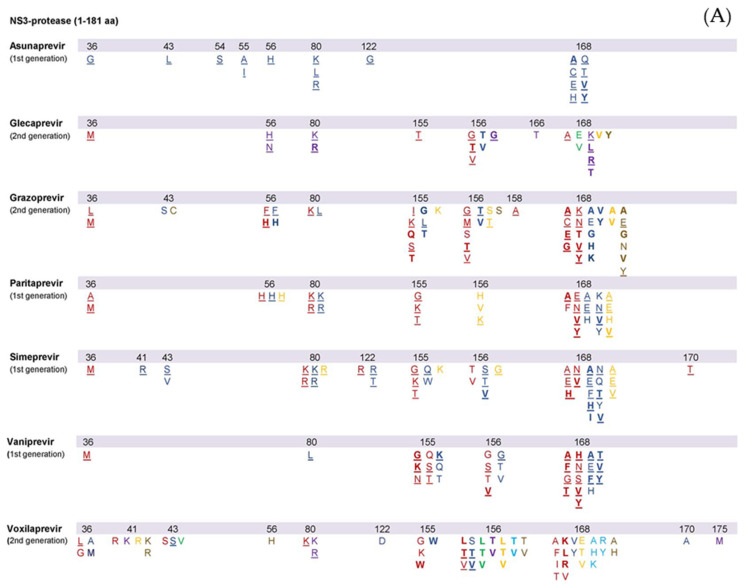

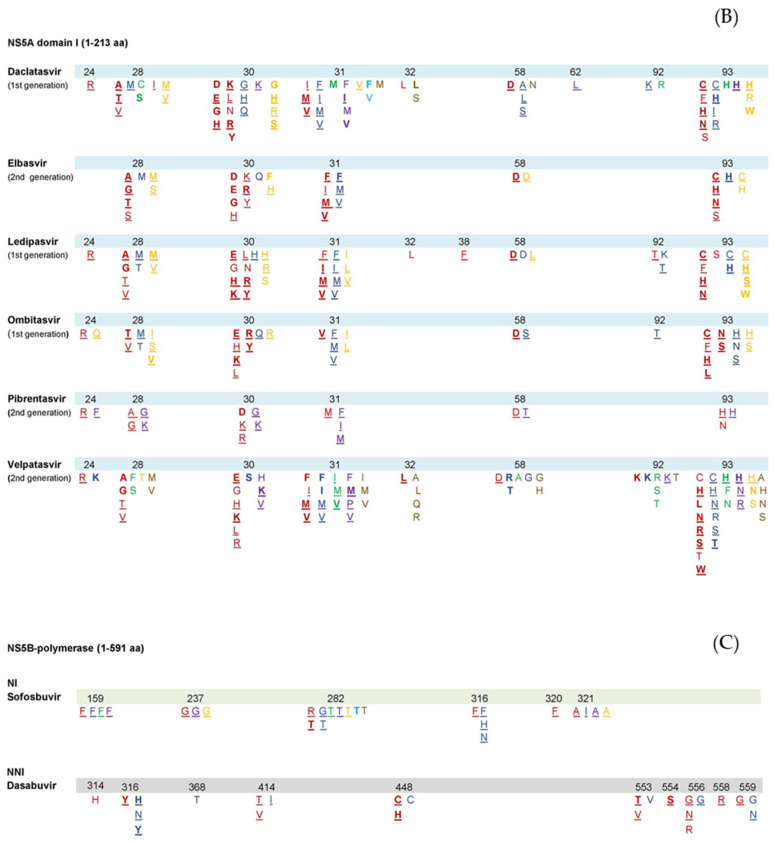
Summary of substitutions associated with resistance to protease inhibitors (**A**), NS5A inhibitors (**B**), and nucleoside and non-nucleoside NS5 B inhibitors (**C**). HCV genotypes and subtypes are represented by different colors: 1a–red, 1b-blue, 2a/b/c–green, 3a–purple, 4a/d–yellow, 5–light blue, 6–brown. Amino acid substitutions detected in vivo in DAA failing patients are underlined, independently of in vitro data information.

First-generation PIs, such as vaniprevir, simeprevir, paritaprevir and asunaprevir, demonstrate increased antiviral potency. However, their genotypic coverage is suboptimal due to a limited genetic barrier to resistance and substantial cross-resistance at various amino acid locations [28]. Second-generation PIs, such as glecaprevir, grazoprevir and voxilaprevir, are more efficacious even in the presence of viral resistance, with a broader genotypic spectrum [28]. Yet, GT3 remains a challenge with PIs, possibly due to certain active polymorphisms [29]. Among second-generation PIs, grazoprevir demonstrates increased activity with 0.2 nM EC50 values against GT1, but its potency decreases in GT3 [29,30]. On the other hand, glecaprevir activity is maintained in GT3 (EC50 in stable GT-3a HCV-replicon = 1.6 ± 0.49 nM, vs. 0.85 ± 0.15 nM in GT-1a and 0.94 ± 0.35 nM in GT-1b) [31]. The list of all known NS3 protease RASs observed both in vitro and in vivo was recently reviewed [15,17,28,32] and is presented in Figure 2A. Variants with RASs in the NS3 protease selected by DAA therapies tend to disappear within months after stopping treatment. There is uncertainty as to whether physicians should wait until the disappearance of RVs before initiating re-treatment with an NS3-4A protease inhibitor regimen in case of DAA failure due to NS3 RAS and whether RASs that were initially selected and then disappeared affect retreatment with NS3-4A protease inhibitors [15].

### 3.1. Simeprevir, SIM

In genotype 1 patients, simeprevir demonstrates increased cross-resistance with other first generation NS3 protease inhibitors [33]. In patients with GT1a, R155K/G/T, V36M, S122R, Q80K/R and D168A/E/H/V substitutions have been linked to treatment failure. Furthermore, substitutions at position 122 (S122R/T) as well as 168 (D168A/E/F/H/N/T/V) seem to play a significant role in treatment outcomes. The available information on GT4 failures is limited, but RASs at the same amino acid locations seem to be involved. In GT1a, b and GT4 failing patients, RASs at several NS3 positions including 43, 80, 122, 155 and 168 were frequently detected [33,34,35]. Regarding the Q80K polymorphism, the data indicate that it downregulates simeprevir in GT1a patients with cirrhosis treated with simeprevir plus sofosbuvir for 12 weeks.

### 3.2. Asunaprevir, ASV

Mutations at position 168 (D168A/E/H/Q/T/V/Y) were observed in both GT1b replicons and in GT1b patients not responding to treatment with asunaprevir [36,37]. Mutations at position S122 were also detected; however, due to their role as natural polymorphisms, their importance in the context of asunaprevir-failure remains unclear. In a phase 2 clinical trial, four treatment naïve, GT1a patients treated with asunaprevir and daclatasvir had the Q80K/L polymorphisms at baseline: three of the patients experienced virological failure. Asunaprevir is not currently recommended for GT1a, yet the contributary effect of Q80K was highlighted in the context of the HALLMARK DUAL trial [37].

### 3.3. Grazoprevir, GZR

Through its different binding with the NS3 catalytic triad [38], grazoprevir is considered a second-generation protease inhibitor efficacious against multi drug RASs such as T54A/S, V36A/M, R155K/Q/T, T54S + R155K or V36M + R155K [39]. NS3RASs commonly related to grazoprevir failure were V36L/M, Y56F/H, Q80K/L, R155I/K/L/S, A156G/M/T/V, V158A and D168A/C/E/G/K/N/V/Y mainly in GT1a, but also in GT1b [40]. RASs located at D168 were detected in GT4 and GT6 failing patients [41]. Among the RAS patterns detected in GT1a failing-patients, Y56H + D168A/N and V36M + A156T demonstrated increased resistance in vitro [40]. Existing data do not support a role of NS3RASs in the downregulation of grazoprevir activity [40].

### 3.4. Paritaprevir/r, PTV

RASs at various positions have been linked with paritaprevir failure. RAS at position 168 demonstrated an increased fold change for paritaprevir activity in vitro and is frequently detected in GT1a-1b and GT4 failing patients [34,42,43,44]. In addition, RASs combinations such as Y56H+D168V and V36M+Q80K+R155K were observed in GT1a-1b and GT4 patients failing PTV-containing treatments [44]. The RAS combination of D168A/V/Y and Y56H produced an additional decrease in PTV activity compared to single D168 substitutions in GT1a-1b and GT4 replicons [35,44]. 

### 3.5. Voxilaprevir, VOX

Voxilaprevir is an EMA and FDA-approved second-generation PI with a broad-spectrum genotypic activity. A156T/V were the most frequently observed substitutions in GT1a and GT1b patients failing VOX treatment [28]. Up until recently, no RASs have been detected in GT2 and GT4 failing-patients. Recent data have described some RASs with increased in vitro fold-change in VOX activity across GTs1–4 [28].

### 3.6. Glecaprevir, GLE

Glecaprevir is another second-generation PI, approved by both the EMA and FDA. It remains efficacious in vitro against several RASs that are known to impair first generation PI efficacy, especially at positions 170, 155 and 54 in GT1 replicons [45]. Nevertheless, substitutions at position 156 lead to increased resistance in GT1a-1b and GT3 replicons [45]. RASs patterns, at certain locations (56 + 168, or 89 + 156, or 156 + 168) demonstrated high levels of resistance, in GT1a, and/or GT1b and/or GT3 replicons [45,46]. In cases of GLE-failure, the most frequently observed NS3 RASs in GT1a and/or GT3 were Q168A/K/L/R, V36 M, Q80 K/R Y56H/N, A156G/T/V and R155T [28,46].

## 4. NS5A Inhibitors

NS5A inhibitors bind to domain I of the NS5A protein, thus downregulating hyperphosphorylation and preventing its dimerization [47]. By acting against NS5A, these agents interfere with several stages of the HCV life cycle: they prevent replication complex formation, decrease virion congregation and release and promote viral degradation [48]. NS5A inhibitors exhibit an intermediate resistance barrier, with rapid selection of RASs within the linker region (aa 28–93). NS5A RAS occur commonly following DAA exposure, can persist for years and increase replication fitness [49]. Resistance to NS5A inhibitors is clinically relevant as this drug class is part of every DAA regimen and can pose a challenge for retreatment [50]. The most frequently detected NS5A RASs in patients with treatment failure involved positions 24, 28, 30, 31, 58, 92 and 93 but with varying impact and prevalence among the various HCV genotypes [15,20,32]. The most significant RAS for NS5A inhibitors in terms of clinical relevance is Y93H. NS5A RVs are long-lasting as opposed to NS3 RVs which seem to disappear within weeks to months [15,40]. Similarly to complex NS3 RAS patterns, complex NS5A RAS patterns confer a higher resistance compared to single RASs and are commonly detected in patients with treatment failure, thus limiting the use of NS5A inhibitors as second-line agents [20]. The list of known NS5A RASs either in vitro or in vivo was recently reviewed in [15,17,28,32] and is presented in Figure 2B. 

### 4.1. Daclatasvir, DCV

Several RASs have been linked to treatment failure in patients receiving daclatasvir. These include H58D, M28A/T, L31I/M/V, Q30E/H/K/R and Y93C/H/N in GT1a; Y93C/H/I/R and L31F/M/V in GT1b; L31I, Y93H and A30K in GT3; and L30H/R/S, L28M/V in GT4a and 4d [28,51,52,53]. The Y93H substitution is a commonly detected RAS in daclatasvir-failing patients [18] and is detected across various genotypes. Natural NS5A RASs have been linked with reduced rates of SVR in GT3 patients [54], as well as GT1a and GT4 patients treated solely with daclatasvir-containing regimens [54]. In contrast to single RASs, complex RAS patterns that involve positions 31 and 93 in GT1b patients can promote increased resistance [28]. RASs at position 30, in combination with one at positions 28, 31, 58, 62 or 93, produced an over 10,000-fold change in the activity of daclatasvir in GT1a (18). Complex RASs patterns involving position 30 have been detected in GT2-5.

### 4.2. Elbasvir, EBR

In patients with elbasvir failure, the most commonly detected RASs were L31F/I/M/V, H58D, M28A/G/T/S, Y93C/H/N/S and Q30H/K/R/Y in GT1a; Y93H and L31F/M/V in GT1b; P58D, Y93C, L30H and L28M/S in GT4a [40,54,55,56]. All RAS patterns were able to produce an increased resistance in GT1a patients (over 1000-fold), while L31M + Y93H significantly impaired EBR efficacy in GT1b patients (5000-fold reduction) [28]. NS5A RASs at positions 28, 30, 31 and 93 induced lower rates of SVR in GT1a patients treated with EBR/GZR for 12 weeks [40,54]. Extension of therapy to 16/18 weeks with the addition of ribavirin resulted in 100% SVR12 rates in patients with baseline RASs [40,54].

### 4.3. Ledipasvir, LDV

Y93H was the most frequently detected RAS in patients who failed ledipasvir-containing treatment. Several LDV RASs have been found in GT1a (K24R, Q30E/H/K/R/Y, M28A/T/V, S38F, L31F/I/M/V, H58D, Y93C/F/N, A92T), GT1b (A92T, L28M, L31I/F/M/V Q30H, Y93C) and GT4 (L30H/R, T/P58L, M31L/V, Y93C/S, L28M) [20,28,51,57,58,59]. Natural NS5A RASs reduce SVR12 rates in GT1a patients treated with LDV/SOF [20]. Prolongation of therapy to 24 weeks and/or addition of ribavirin yielded increased SVR rates [32]. Natural RASs at positions 28 and 30 in GT4 patients also reduced SVR12 rates [57].

### 4.4. Ombitasvir, OMB

The RASs with the greatest impact in ombitasvir-treated patients were Q30E/H/K/L/R/Y, M28T/V, Y93C/F/H/L/N/S, L31V and H58D in GT1a; L31F/M/V, Y93H/S, L28M and R30Q in GT1b and L28S/V in GT4a or 4d [28,34,60]. In patients with GT1b genotype, RAS involving positions 31 and 93 resulted in an increased resistance, similar to patterns involving positions 30 and 93 in GT1a patients [28,60]. The RAS pattern L28V+T58S, detected in GT4 patients failing treatment with ombitasvir, resulted in over a 500-fold change in resistance [60].

### 4.5. Pibrentasvir, PIB

Pibrentasvir (PIB) is a second-generation NS5A inhibitor with a broad-spectrum genotypic activity, recently approved by the EMA and FDA. Data regarding pibrentasvir resistance are currently limited. RASs detected in GT3 patients failing pibrentasvir treatment included A30G/K, P58T, S24F, M28G/K, Y93H and L31F/I/M, while GT1a patients relapsed with one or more of these substitutions: M28A/G, K24R, L31M, Y93H, Q30K/R and H58D [28,61,62,63]. Natural NS5A RASs such as A30K and Y93H have been directly linked to reduced rates of SVR in GT3patients treated with PIB/GLE for 12 weeks, whereas the presence of natural RASs had no apparent effect in other genotypes [63].

### 4.6. Velpatasvir, VEL

Velpatasvir (VEL) is another second-generation NS5A inhibitor with enhanced genotypic activity, approved by both the FDA and EMA. NS5A RASs related to VEL-failure involve positions 24, 28, 30, 31, 32, 58, 92 and 93. In patients with velpatasvir failure, the most commonly detected RASs were Q30E/H/K/L/R, M28T/V, Y93H/N/R/S/W and L31I/M/V in GT1a patients; Y93C/H/N/S/T and L31M/V in GT-1b; L31I/M/V and Y93H in GT2; and A30K/V, E92K, Y93H/N/R and L31M/P/V in GT3 [28,64,65,66]. The RAS pattern Q30H+Y93H in GT1a patients was associated with increased levels of resistance [28,64]. In the ASTRAL-3 trial, natural NS5A RASs were found in 43/274 (16%) GT3 patients, of whom 88% reached SVR at week 12, compared to 97% of the 231 patients with undetectable natural NS5A RASs. Of note, among 25 patients with natural Y93HRAS at baseline, only 21 (84%) achieved SVR at week 12 [65,66].

## 5. NS5B Polymerase Inhibitors

The RNA polymerase NS5B is structured in a “right-hand motif”, consisting of finger, palm and thumb domains. NS5B inhibitors include both nucleotide (NIs) and non-nucleotide inhibitors (NNIs). NIs act as NS5B substrates, occupying the NS5B active site, thus leading to direct chain termination. Due to the limited variability of the NS5B active site, NIs are efficacious against all HCV genotypes, while maintaining an increased tolerance to resistance [67]. NNIs inhibit NS5B by connecting to different allosteric sites. In contrast to NIs, their efficacy is almost exclusively limited against GT1. Among DAAs, NNIs have the lowest resistance barrier [32]. The list of all known NS5B-polymerase RASs either in vitro or in vivo was recently reviewed in [15,17,28,32] and is summarized in Figure 2C.

### 5.1. Sofosbuvir, SOF

Sofosbuvir has been the backbone of HCV treatment due to its pangenotypic activity, as it targets the conserved active site of NS5B while demonstrating a high barrier to resistance. RAS S282T that is situated close to the enzyme’s active site has been shown in vitro to confer resistance to Sofosbuvir but is rarely found in patients who failed sofosbuvir-containing regimens (<4%) [28]. RAS S282T is a highly unfit substitution that is usually only detectable for very short periods of time [68]. The significance of other RAS in patients who failed sofosbuvir-based regimens, such as the L159F and C316N RAS, is unclear [68,69]. Data deriving from pooled-analyses of clinical trials and observational data highlighted the role of L159F RAS, either alone or in combination with C316N or L320F, in GT1 and GT3 patients with SOF failure [69,70,71]. RAS pattern L159F + C316N was mainly detected in GT1b SOF-failing patients [69,70,71], whereas L159F + V321A was exclusively detected in GT1a and GT3 patients [69,70,71]. Of note, sofosbuvir retreatment has been linked to higher rates of treatment failure and selection of S282T [69].

### 5.2. Dasabuvir, DSV

NS5B RASs related to dasabuvir treatment failure in GT1a infected patients were Y448C/H, G554S, A553T/V, G558R, C316Y, D559G, S556G/N and M414T/V. RASs in GT1b were M414I, S556G, D559Nand C316H//N/Y [28]. Overall, S556G was the most commonly detected RAS in GT1a and GT1b DSV-failing patients [34].

## 6. Patterns of RASs and Retreatment Options

Until recently, the appearance of RVs had an impact on the efficacy of second and third-line treatment options [15,16,30,32,34]. The utility of RAS testing is directly linked to both the DAA regimen and the patient’s characteristics [16]. However, the availability of new drug combinations with broad-spectrum genotypic activity, a higher barrier to resistance and antiviral potency may change the debate. Nevertheless, until newer DAAs become widely available and the obstacle of resistance is tackled, the HCV genotypic resistance testing will remain the cornerstone of personalized treatment, particularly in the context of DAA failure [16]. Outside the US, the use of HCV resistance testing is mitigated by the absence of well-studied, validated and easily accessible methods. Furthermore, given that the prevalence and impact of different RASs varies with the viral profile (genotype and subtype), the extrapolation of conclusions based on resistance testing becomes challenging and is only partially supported by the available algorithms/databases [14], reviews [15,32,35] and guidelines [16,17].

Of note, 57% of GT1a, 93% of GT1b and 100% of GT4 patients who failed an NS5A inhibitor had no RAS in NS3 and were able to receive PI-based retreatment. Vice versa, 86% of GT1a and 83% of GT1b PI-experienced individuals had no NS5A RASs and, thus, were suitable to receive NS5A inhibitor-based treatment. The short half-life of NS3 RASs in patients with previous PI failure can explain the high SVR rates with retreatment regimens that included a PI plus sofosbuvir without an NS5A inhibitor. A study of 220 patients who failed DAA therapy from 39 hospitals in Spain showed that 88.6% had one or more RAS. The subtype-specific pattern of RAS emergence underscores the importance of accurate HCV subtyping [72]. The Q80K substitution in NS3, Y93H in NS5A and L159F in NS5B was common to all subtypes. A total of 18.6% did not have a substitution in the target regions. Ribavirin did not seem to have an effect on the emergence or frequency of RAS-associated variants.

In everyday clinical practice, most patients that experienced DAA failure were treated with sofosbuvir plus a first-generation NS5A inhibitor. NS5A RASs patterns (either typical or diverse) were detected based on the NS5A inhibitor and the viral geno/subtype [18]. In GT1a patients, Q30H/R variants were detected after treatment failure with ledipasvir or daclatasvir plus sofosbuvir. Y93H variants played a major role only for ledipasvir/sofosbuvir, while their importance in the context of daclatasvir/sofosbuvir was limited. The use of second-generation NS5Ainhibitors including pibrentasvir and velpatasvir [73,74] or the combination of elbasvir, grazoprevir, sofosbuvir and ribavirin are feasible retreatment options [75]. In patients who failed treatment with daclatasvir/sofosbuvir, the absence of the Y93H variant provides the option of retreatment with ledipasvir or ombitasvir [18]. The three-drug (3D) regimen (ritonavir-boosted paritaprevir, ombitasvir and dasabuvir) led to the selection of R155K in NS3 of GT1a and D168V in GT1b, which was detected frequently in combination with Y56H, enhancing the resistance level. This resistance profile is similar to that of simeprevir, but Y56H exclusively occurred after paritaprevir administration. Within GT1a NS5A, M28T/V and Q30H/R were mainly detected and M28 variants are characteristic for ombitasvir treatment. Especially for 3D failures, retreatment options used to be limited due to the increased number of RASs and the overlapping resistance profiles [18]. Novel treatment combinations, such as sofosbuvir/grazoprevir/elbasvir/ribavirin [75] or sofosbuvir/velpatasvir/voxilaprevir [73,74] or even sofosbuvir plus glecaprevir/pibrentasvir, demonstrate increased efficacy in DAA-experienced patients even in the presence of RASs [18,76].

In GT1b patients, similar NS5A RASs patterns were detected for daclatasvir and ledipasvir and, compared to GT1a patients, Y93H was highly prevalent. Since Y93H variants persist long-term and are associated with resistance to ombitasvir, for GT1b retreatment, the combination of a PI with a second-generation NS5A inhibitor is a good alternative. Y93H was the major RAS for ombitasvir in GT 1b and subsequently for all first-generation NS5A inhibitors. NS5B non-nucleoside RASs were rare in GT1a, but in GT1b, S556G was selected in combination with C316N.

Treatment failure with daclatasvir/sofosbuvir in GT3 patients was linked to a strong increase in Y93H. Y93H on the other hand was not detected after ledipasvir/sofosbuvir treatment in the same genotype, possibly because of the limited antiviral efficacy of ledipasvir in GT3 [18]. Second-generation NS5A inhibitors demonstrated increased antiviral activity against GT3 isolates harboring Y93H, while recently GT3-sensitive PIs were approved. Although SVR rates after initial treatment with pibrentasvir/glecaprevir or retreatment with velpatasvir/sofosbuvir/voxilaprevir were 97% and 95% respectively, most patients who failed treatment were GT3 [73,74]. Treatment failure was not associated with a specific RAS. It is therefore evident that retreatment of GT3 patients remains challenging. The addition of ribavirin in retreatment regimens may provide some benefit in difficult-to-treat GT3 patients.

In patients with GT4 infection, NS5A L28M followed by Y93C/H/S were the main RASs linked to daclatasvir/sofosbuvir and ledipasvir/sofosbuvir treatment failure, respectively. Notably, the moderate frequency of Y93 variants allows for retreatment with velpatasvir/sofosbuvir, as these variants produce low levels of velpatasvir resistance. Typical RASs detected after two-drug (2D-ombitasvir, paritaprevir and ritonavir) failure in GT4 were L28V in NS5A and D168V in NS3, while Y93 variants were detected less frequently.

Voxilaprevir/velpatasvir/sofosbuvir as multiple targeted rescue therapy is the first choice for patients who fail DAA therapy with rates of SVR above 90%, irrespective of the presence of RAS [77]. Despite the high efficacy of the fixed triple combination, treatment failure has been observed in patients with genotypes 1a and 3 and cirrhosis [78]. Treatment failure was not associated with a specific RAS. A substitution in position 156 of NS3 has been recently associated with resistance to glecaprevir or voxilaprevir in genotypes 1–4 (but not for genotypes 5 and 6) [79]. A cohort of 144 patients who failed DAA therapy received retreatment with sofosbuvir/ velpatasvir/ voxilaprevir [80]. Overall SVR rate was 90% but was significantly lower in GT3 (81%). GT3 and presence of cirrhosis correlated with treatment failure. Pre-treatment RAS was not associated with response to sofosbuvir/velpatasvir/voxilaprevir.

A study of 177 GT1 patients who failed previous treatment with sofosbuvir and a NS5A inhibitor showed high SVR rates with retreatment with glecaprevir/pibrentasvir [81]. NS5A RAS were detected in 75% of GT1a patients at baseline. SVR12 was lower in those with double-linked (88%) and triple-linked (63%) NS5A substitutions compared with patients without NS5A baseline substitution (97%) and those with single substitutions (93%). No specific RAS were associated with lower SVR. Supplementary Table S1 highlights retreatment options in DAA failure.

The European Society for the Study of the Liver (EASL) recommends that patients without cirrhosis or with compensated cirrhosis who failed after a DAA-containing regimen should be retreated with the fixed-dose combination of sofosbuvir/velpatasvir/voxilaprevir for 12 weeks. Patients who have predictors of lower response (advanced liver disease, multiple courses of DAA therapy, complex NS5A RAS profile) can be retreated with the combination of sofosbuvir plus glecaprevir/pibrentasvir for 12 weeks. In patients with NS5A RASs who failed twice or more to achieve SVR, the triple combination of sofosbuvir/velpatasvir/voxilaprevir, or the triple combination of sofosbuvir with glecaprevir/pibrentasvir can be administered for 12 weeks with weight-based ribavirin and/or treatment duration can be prolonged to 16–24 weeks. Patients with decompensated (Child-Pugh B or C) cirrhosis have a contraindication for the use of protease inhibitors and should be retreated with sofosbuvir/velpatasvir with weight-based ribavirin for 24 weeks [12].

## 7. Conclusions

Several DAA-based regimens are available for the treatment of chronic HCV infection. Despite their high efficacy, some patients demonstrate treatment failure which has been linked to the detection of RASs at baseline. In patients naïve to DAAs, RAS frequencies within the viral population either as transmitted or natural polymorphisms alongside several treatment options allow for a RASs-free treatment for all HCV genotypes. The presence of NS3 Q80K or NS5A RASs at baseline was linked to a reduced efficacy for several DAAs; however, this was overcome with intensified treatment schemes, such as the prolongation of treatment and/or the addition of ribavirin [54]. For DAA-experienced patients, retreatment options based on the RASs-free approach are limited. Resistance profiles based on the viral genotype and subtype are currently available through the analysis of a larger number of patients who failed DAA treatment, thus allowing for qualitative/quantitative comparison and comparative approaches. Since second generation and broad-spectrum regimens are not widely available, reports on the efficacy of rescue treatments based on the resistance profile of patients who failed DAAs are of great significance. This approach has been associated with SVR rates of more than approach 90% [18,76].

In summary, the identified resistance patterns for broadly used DAA regimens allow for the selection of specific retreatment options based on the result of resistance analysis. Resistance profiles for GT1b, more than the GT1a in Europe and 3a-infected patients largely overlap, thus limiting individualized RASs-free retreatment options considerably.

## Data Availability

Not applicable.

## Supplementary Materials

The following are available online at https://www.mdpi.com/article/10.3390/v13102096/s1, Table S1: Retreatment options in DAA failure.
