# Supplementary material for: The Role of RASs /RVs in the Current Management of HCV
Source: Viruses. 2021 Oct 18;13(10):2096. doi: 10.3390/v13102096 (PMC8539246; doi:10.3390/v13102096)
Supplement: Supplementary file 1 [file viruses-13-02096-s001.zip › viruses-1299046-supplementary.pdf]

Table S1. Retreatment options in DAA failure.

**Genotype**	**Regimen failure**	**Retreatment options**
GT1a	Daclatasvir/sofosbuvir<br>Ledipasvir/sofosbuvir	Sofosbuvir/velpatasvir<br>Glecaprevir/pibrentasvir<br>Sofosbuvir/velpatasvir/voxilaprevir<br>Sofosbuvir/grazoprevir/elbasvir/ribavirin
	Ritonavir-boosted paritaprevir, ombitasvir, and dasabuvir (3D)	Sofosbuvir/grazoprevir/elbasvir/ribavirin<br>Sofosbuvir/velpatasvir/voxilaprevir<br>Sofosbuvir plus glecaprevir/ pibrentasvir
GT1b	Daclatasvir/sofosbuvir<br>Ledipasvir/sofosbuvir	PI with a second-generation NS5A inhibitor<br>Sofosbuvir/velpatasvir/voxilaprevir
GT3	Daclatasvir/sofosbuvir	Glecaprevir/pibrentasvir<br>Sofosbuvir/velpatasvir/voxilaprevir<br>(With ribavirin in difficult-to-treat cases)
GT4	Daclatasvir/sofosbuvir<br>Ledipasvir/sofosbuvir<br>Ritonavir-boosted paritaprevir, ombitasvir (2D)	Sofosbuvir/velpatasvir<br>Sofosbuvir/velpatasvir/voxilaprevir
